# Surface Plasmon Resonance Enhanced Photoelectrochemical Sensing of Cysteine Based on Au Nanoparticle-Decorated ZnO@graphene Quantum Dots

**DOI:** 10.3390/molecules29051002

**Published:** 2024-02-25

**Authors:** Jiaxin Liu, Fancheng Lin, Yan Wang

**Affiliations:** College of Chemistry, Chemical Engineering and Materials Science, Shandong Normal University, Jinan 250014, China; sdnuljx@163.com (J.L.); sdnulfc@163.com (F.L.)

**Keywords:** core–shell quantum dots, graphene, ZnO, Au nanoparticles, cysteine, photoelectrochemical biosensor

## Abstract

In this work, Au nanoparticle-decorated ZnO@graphene core–shell quantum dots (Au-ZnO@graphene QDs) were successfully prepared and firstly used to modify an ITO electrode for the construction of a novel photoelectrochemical biosensor (Au-ZnO@graphene QDs/ITO). Characterization of the prepared nanomaterials was conducted using transmission electron microscopy, steady-state fluorescence spectroscopy and the X-ray diffraction method. The results indicated that the synthesized ternary nanomaterials displayed excellent photoelectrochemical performance, which was much better than that of ZnO@graphene QDs and pristine ZnO quantum dots. The graphene and ZnO quantum dots formed an effective interfacial electric field, enhancing photogenerated electron–hole pairs separation and leading to a remarkable improvement in the photoelectrochemical performance of ZnO@graphene QDs. The strong surface plasmon resonance effect achieved by directly attaching Au nanoparticles to ZnO@graphene QDs led to a notable increase in the photocurrent response through electrochemical field effect amplification. Based on the specifical recognition between cysteine and Au-ZnO@graphene QDs/ITO through the specificity of Au-S bonds, a light-driven photoelectrochemical sensor was fabricated for cysteine detection. The novel photoelectrochemical biosensor exhibited outstanding analytical capabilities in detecting cysteine with an extremely low detection limit of 8.9 nM and excellent selectivity. Hence, the Au-ZnO@graphene QDs is a promising candidate as a novel advanced photosensitive material in the field of photoelectrochemical biosensing.

## 1. Introduction

Cysteine, a crucial amino acid-containing thiol group, serves significant functions in numerous biological processes. Cysteine participates in tissue protein synthesis, posttranslational modifications and the construction of active sites in certain enzymes [1]. It was found that diseases such as acquired immune deficiency syndrome, liver injury and Alzheimer’s disease were accompanied by cysteine deficiency [2]. Cysteine levels in the body have been acknowledged as a significant marker for diagnosing diseases [3]. Hence, it is crucial to investigate the functions of cysteine in cells and diagnose diseases though its sensitive and selective detection.

Numerous techniques have been reported to monitor cysteine, including us of a colorimetric sensor [4], the HPLC method [5], capillary electrophoresis analysis [6], spectrofluorimetry [7], electrochemical methods [8,9] and photoelectrochemical (PEC) detection [10,11]. Among the different methodologies used, photoelectrochemical measurements have attracted considerable interest because of their excellent analytical properties. In contrast to electrochemical analysis, photoelectrochemical detection exhibits a significantly reduced background as a result of the separation between excitation light sources and detection photocurrent signals. Therefore, this method demonstrates encouraging analytical uses in the fields of bioanalysis, environmental monitoring and medicine detection [12,13]. Despite the mentioned advantages, the development and application of high-performance photoactive materials are still urgent issues to be solved in photoelectrochemical sensing research.

The research community has shown significant interest in the utilization of graphene– inorganic semiconductor composites for photocatalysis and photoelectrochemical detection in the past few years [14,15]. Researchers have reported that the nanocomposites exhibited significant enhancement in photocatalytic performance when large-area monodispersed graphene combined with TiO_2_ [16,17], ZnO [18,19], CdS [20], ZnFe_2_O_4_ [21], etc. In the heterostructures, inorganic semiconductor materials served as substances that absorb light and produce charge carriers, whereas the graphene layer played the role of a superb conductive framework, owing to its exceptional electron mobility. The enhanced PEC property was ultimately achieved due to the significant facilitation of photogenerated electron migration to the electrode and the efficient strengthening of charge separation [22].

Recently, ZnO/graphene hybrid nanocomposites have attracted significant research attention in various applications, such as gas sensors [23,24] and electrochemical sensors [25]. Due to the combined effect of the two materials and the additional graphene function, the ZnO/graphene composite material enhances the overall electrochemical performance and stability of a sensor, with a fast response and a satisfactory sensing performance. The synergistic effect of ZnO/graphene improves binding stability with macromolecules, making it a potential candidate for gas sensor device design. Zhang et al. developed a novel ZnO/graphene gas sensor for the selective and sensitive detection of NO_2_ gas at low detection limits at room temperature [23]. Niavol et al. successfully synthesized ZnO quantum dots modified on graphene nanoplatelets (GNP) using a simple hydrothermal method [24]. The gas sensing performance of the developed sensor based on ZnO/GNP for ethylene glycol is significantly enhanced. ZnO nanorods can effectively form a three-dimensional (3D) structure with graphene. The produced hybrid nanocomposite (ZnO@G) exhibits an extremely large surface area and high conductivity, which have been employed in electrochemical sensor [26,27,28,29]. ZnO@G nanocomposites have been synthesized and used for electrode surface modification to detect glucose [26], ascorbic acid, dopamine, and uric acid [27] in a recent report. Through colloidal agglomeration effect, Yukird et al. synthesized ZnO@G nanocomposite materials to modify electrodes and simultaneously measure Cd^2+^and Pb^2+^ [28]. Shen’s team constructed a sensing material of a Ni-doped ZnO/graphene nanosheets composite using a simple hydrothermal method, which was further used for electrochemical determination of acetaminophen and 17β-estradiol [29]. The study provides a new method for designing high-performance sensing materials and exploring their applications in trace drug determination.

ZnO nanomaterial is a kind of attractive photoactive material because it displays low cost, non-toxicity and effective photoelectric properties. Nonetheless, pure ZnO typically has low efficiency in capturing light and separating electrons and holes [30,31]. ZnO/graphene hybrid nanocomposites have recently attracted great interest in photocatalysts [32] and photoelectrochemical sensors [33,34]. Liu’s team has developed a PEC aptamer sensor based on a ZnO/graphene composite and S6 aptamer for an SK-BR-3 cancer cell assay [33]. Yan et al. proposed a simple heat treatment method to prepare ZnO/graphene-sensitized structures. A signal-off PEC sensor for adenosine triphosphate (ATP) detection was designed based on synthesized nanocomposites as photoactive materials and ATP binding aptamers as recognition elements [34]. Nevertheless, the optimum photocatalytic performance of ZnO/graphene hybrid material cannot be attained because the graphene–ZnO semiconductor interface has a relatively small contact area and a low heterojunction electric field. Recently, a simple solution method was reported to synthesize core–shell quantum dots called ZnO@graphene quantum dots, which involved a ZnO quantum dot core enveloped by a single-layer graphene shell [35]. This emissive hybrid quantum dot was applied to develop a light-emitting diode with satisfactory results. Bu et al. further optimized the preparation conditions of the novel nanocomposite and studied its PEC properties and photocatalytic performance [36]. The investigations demonstrated that ZnO@graphene quantum dots exhibited significantly enhanced PEC performance in comparison to those of the ZnO/graphene composite with the structure of a large-area graphene-loaded ZnO because three-dimensional nanocoating core@shell structure can form large contact area and strong effective interfacial electric field which promoting the charge separation. Therefore, this new type of core@shell nanostructure material has valuable application potential in solar cells and photocatalysis. However, to the best of our knowledge, this photoactive material has never been exploited for photoelectrochemical sensing detection.

Another crucial aspect of the PEC system design is the light absorption of the photoelectrode materials. To enhance the absorption of visible light by metal oxide semiconductors, noble metals such as Au can be incorporated, taking advantage of their surface plasmon resonance (SPR) characteristic [37]. In this work, Au nanoparticle-sensitized ZnO@graphene core–shell quantum dots (Au-ZnO@graphene QDs) were successfully prepared through simple synthesis and modification methods. The results indicated that this novel ternary nanomaterial displayed an excellent photoelectrochemical performance, which was much better than that of pure ZnO quantum dots and ZnO@graphene QDs. Attaching Au nanoparticles directly to ZnO@graphene QDs provides a robust surface plasmon resonance effect, resulting in a significantly enhanced photocurrent response through electrochemical field effect amplification. Therefore, we developed an innovative PEC biosensing platform using Au-ZnO@graphene QDs ternary nanocomposites, which exhibited an enhanced photocurrent response to cysteine oxidation. The prepared biosensor exhibited excellent sensitivity, extensive linearity, exceptional selectivity, and notable reproducibility in detecting cysteine. The photoelectrochemical sensing method was successfully employed to quantify cysteine levels in both human serum and urine.

## 2. Results and Discussion

### 2.1. Characterization of the Prepared Nanomaterials

**Figure 1:**
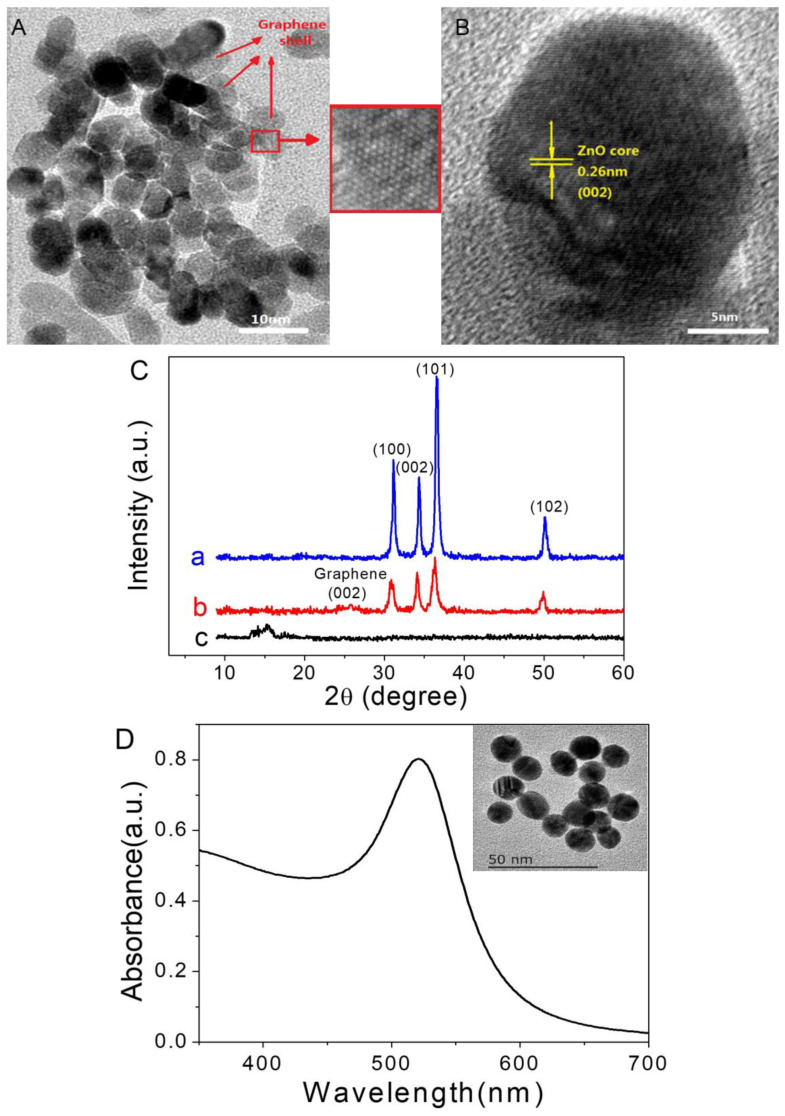
(**A**) TEM images of ZnO@graphene QDs; (**B**) HRTEM images of ZnO@graphene QDs; (**C**) XRD spectra of (a) ZnO QDs, (b) ZnO@graphene QDs, and (c) graphene oxide; (**D**) UV–vis absorption spectra and the TEM morphology (inset) of gold nanoparticles.

Figure 1A,B display the transition electron microscopy (TEM) morphologies of ZnO@graphene QDs. The TEM image under low magnification in Figure 1A exhibits that nanoparticles with an average size of approximately 15 nm are uniformly distributed. The red arrows point out the outer graphene shell. The magnified HRTEM image captured from the layer encircling ZnO QDs is displayed in the close red squared region, in which a hexagonal atomic lattice with uniform contrast can be clearly distinguished. In addition, the distance between carbon atoms was measured to be approximately 0.14 nm, indicating that the layer is composed of monolayer graphene. The HRTEM image of one ZnO@graphene QD (Figure 1B) clearly displayed the (002) crystal plane of ZnO. The above results demonstrate that this hybrid quantum dot which possesses core–shell structure, is comprised of a ZnO core enveloped by a single-layer graphene shell.

The X-ray diffraction technique was employed to investigate the composition and structure. The crystal structure of the ZnO QDs (Figure 1C curve a) showed the characteristic diffraction peaks of wurtzite ZnO at 31.8°, 34.1°, 36.4° and 49.8°, which were related to the (100), (002), (101) and (102) planes (JCPDS 36-1451) [30]. In contrast, the XRD diffractograms of ZnO@graphene QDs exhibited all the diffraction peaks of ZnO QDs and showcased an additional wide and intense peak at 25.8° (curve b). This peak corresponds to the (002) crystal plane of graphene [22], suggesting the successful modification of graphene on ZnO QDs. Moreover, the diffraction peaks of ZnO did not change after coating the graphene, indicating that the crystal lattice of ZnO is not doped by graphene and its crystal structure is not influenced. Furthermore, the diffraction peak (14.8°) of GO is not observed in curve b, demonstrating that graphene layer in the hybrid quantum dot has a high degree of reduction [14]. The above result further proved that the ZnO core was covered with a graphene shell to form a core@shell structure.

Figure 1D presents UV–vis absorption spectra and TEM morphology of Au nanoparticles. AuNPs with an average size of about 13 nm exhibited an absorption maximum at 520 nm, implying that AuNPs with good monodispersity can efficiently absorb the visible light [38].

### 2.2. Optical Property of the Prepared Nanomaterials

**Figure 2:**
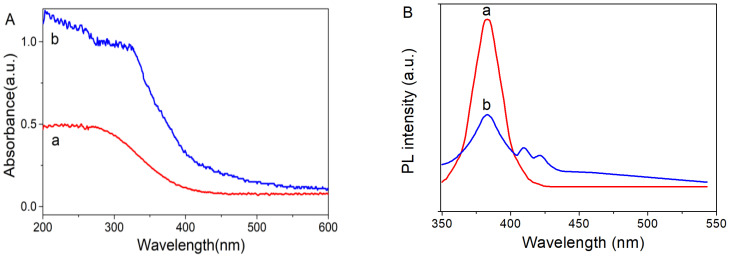
(**A**) DRS spectra of (a) ZnO QDs and (b) ZnO@graphene QDs; (**B**) PL spectra of (a) ZnO QDs and (b) ZnO@graphene QDs.

Steady-state fluorescence spectroscopy (PL) and UV–Vis diffuse reflectance spectroscopy (DRS) were applied for optical performance analysis. The DRS results in Figure 2A demonstrated that the bandgap absorption edge of ZnO QDs was in the ultraviolet region. After coating with graphene, the optical absorption edge of ZnO@graphene QDs was red-shifted to 410 nm, which was attributed to the zero-bandgap structure and the broad spectral absorption capability of graphene. In the whole ultraviolet and visible region addition, ZnO@graphene QDs composite showed stronger absorption intensity than that of ZnO QDs, which revealed that the optical absorption performance of ZnO QDs can be significantly enhanced due to the graphene modification effect.

As displayed in Figure 2B, the 380 nm fluorescence emission peak of ZnO quantum dots is attributed to the electron transition from the valence band to the conduction band in ZnO QDs. The PL spectra of ZnO@graphene QDs (curve b) exhibited two additional emission peaks at 406 nm and 432 nm, owing to the creation of C-O-Zn bonds that connect graphene and ZnO, resulting in the formation of core–shell structures. When ZnO@graphene QDs are excited by light, some electrons enter the lowest occupied molecular orbitals of G–O with an epoxy bond in the graphene shell, resulting in the two additional emission peaks. In addition, PL spectra can reflect the separation efficiency of photogenerated charges in optoelectronic substances [35]. The PL intensity of ZnO@graphene QDs is lower than that of ZnO QDs, suggesting that composite material efficiently suppresses the recombination of electron–hole pairs and possesses outstanding optical properties.

### 2.3. PEC Performance of the Sensor

**Figure 3:**
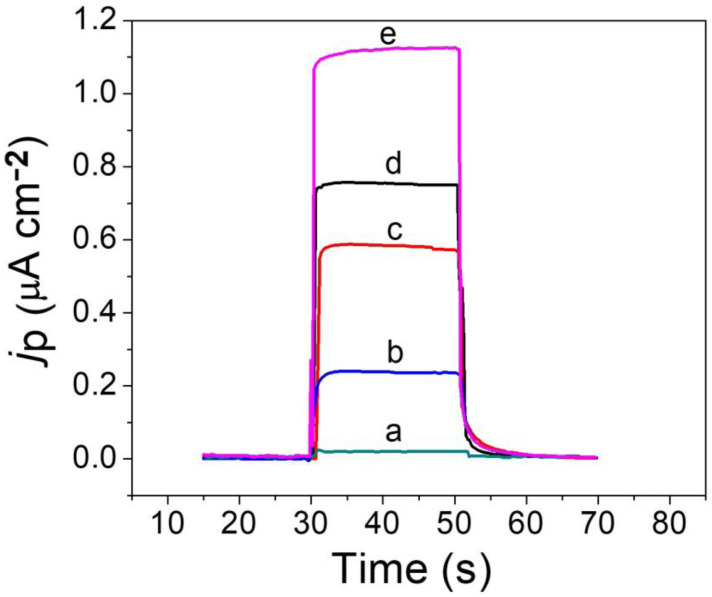
Photocurrent responses of (a) ITO, (b) ZnO QDs/ITO, (c) ZnO@graphene QDs/ITO, (d) Au-ZnO@graphene QDs/ITO in 0.1 M phosphate buffer (pH 7.0) and (e) Au-ZnO@graphene QDs/ITO in 0.1 M phosphate buffer containing 10 μM cysteine at an applied potential of 0.0 V under the irradiation of a xenon light source.

The PEC properties of Au-ZnO@graphene QDs nanocomposites was investigated by measuring the photocurrent density on various modified electrodes in pH 7.0 phosphate buffer under white light irradiation at a working voltage of 0.0 V. The bare ITO electrode had no photocurrent response (Figure 3 curve a). Curve b in Figure 3 exhibited a low photocurrent density of 0.2 μA/cm^2^ on the ITO electrode modified with ZnO QDs, which can be attributed to inadequate light absorption and insufficient separation of electrons and holes. In comparison, ZnO@graphene QDs/ITO exhibited approximately a three times higher photocurrent compared to ZnO QDs/ITO (curve c). This demonstrates that the three-dimensional nanocoating core@shell structure effectively inhibits the recombination of charge carriers, resulting in enhanced photoelectrochemical activity. Following the modification of AuNPs on ZnO@graphene QDs/ITO, the photocurrent signal exhibited an approximately 4-fold increase compared to ZnO QDs/ITO (curve d). The result demonstrated that the direct adhesion of AuNPs to ZnO@graphene QDs provides a robust SPR effect to further improve the optical absorption performance of nanocomposites in the whole visible region, resulting in a significantly enhanced photocurrent response. As seen in curve e, the Au-ZnO@graphene QDs/ITO electrode showed an enhanced photocurrent density of 1.1 μA/cm^2^ when cysteine was present, indicating that cysteine as an electron donor can effectively promote the separation of electron–hole pairs which leads to further ascent of the photocurrent response.

**Scheme 1:**
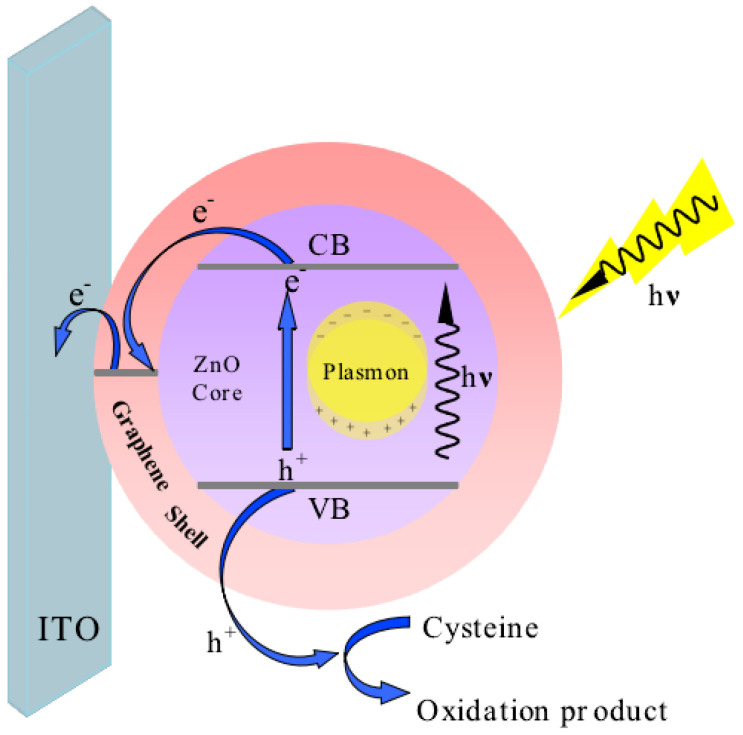
The charge transport pathway and detection principle of PEC cysteine sensor.

A schematic diagram of Au-ZnO@graphene QDs/ITO for cysteine detection is presented in Scheme 1. During the conversion of photocurrents, the electrons on the valence band (VB) of the ZnO are firstly excited to the conduction band (CB) of ZnO to generate electron–hole pairs after absorbing white light. Afterward, the excitation electrons on CB of the ZnO core swiftly transfer to the graphene shell due to well-matched energy levels and subsequently inject into the ITO electrode for the formation of a current in the external circuit. Then, electron donor cysteine in PB captures the separated holes, resulting in the oxidization of cysteine. The consumption of the photogenerated holes by cysteine effectively suppress charge recombination, leading to an enhanced photocurrent response in the existence of cysteine. The improvement of the photoelectrochemical properties of the PEC sensor is attributed to several factors as follows: (1) The three-dimensional nanocoating core@shell structure can form a large contact area and a strong effective interfacial electric field due to the firm and uniform bonding of the ZnO core and graphene shell, promoting charge separation. (2) The graphene shell has excellent conductivity which can bring about rapid charge transport. (3) Because plasmonic Au nanoparticles have excellent light absorption capability and good conductivity, AuNPs-doped ZnO@graphene QDs nanohybrids exhibit enhanced incident light absorption and improved photoelectric conversion efficiency due to the amplified electrochemical field effect by the surface plasmon resonance of AuNPs. (4) During the detection process, cysteine can be selectively captured by the Au-S bond. The specifical recognition function between cysteine and Au-ZnO@graphene QDs/ITO through the specificity of Au-S bonds enhances the selectivity of the sensor. The above charge transfer mechanism further confirms that the Au-ZnO@graphene QDs heterostructure exhibits distinctly enhanced PEC activity contributing to a higher separation efficiency of photoexcited charges, faster migration rates of electrons, and an excellent synergistic effect.

### 2.4. Photoelectrochemical Sensing of Cysteine

The experimental parameters of the PEC sensor for detecting cysteine were optimized to obtain the best performance. The applied detection potential is a vital influence factor for the sensitivity of the sensor. The photocurrent intensity increased with the increase in voltage in the range of −0.3~0.0 V. However, the trend of photocurrent changes with potential tended to be gentle after 0.0 V. In order to eliminate the interference of some coexisting reducing substances, a lower potential is more suitable for the photoelectric detection of cysteine. Therefore, 0.0 V was chosen to be the optimized applied potential to ensure the sensitivity and selectivity of detection.

**Figure 4:**
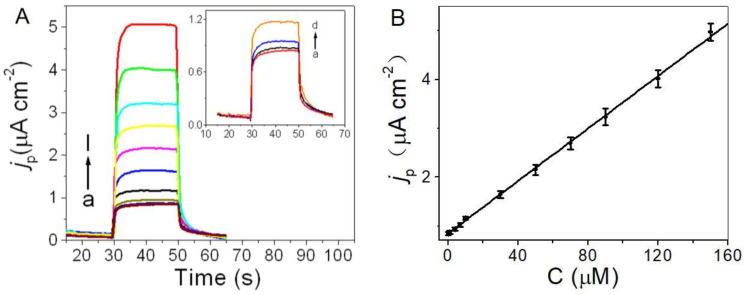
(**A**) Photocurrent response to cysteine at a series of concentrations: (a): 0.1, (b): 0.5, (c): 1.0, (d): 4.0, (e): 7.0, (f): 10.0, (g): 30.0, (h): 50.0, (i): 70.0, (j): 90.0, (k): 120.0, (l): 150.0 μM. Inset: magnified response of (a): 0.1, (b): 0.5, (c): 1.0, (d): 4.0 μM cysteine. (**B**) The linear calibration plot between photocurrent intensity and cysteine concentration.

Figure 4A illustrates the photocurrent responses of the PEC sensor in different concentrations of cysteine under the optimum conditions, in which the photocurrent density gradually increased alone with the concentration. The sensor has a linear response in the 0.1 to 150 μM cysteine concentration range with a detection limit of 8.9 nM based on 3σ/S (Figure 4B). The regression equation was Jp (μA/cm^2^) = 0.8280 + 0.0273c (μM) (R = 0.9993). In comparison to previously reported PEC methods, the PEC sensor described in the present work possesses a wider linear response range and higher sensitivity, demonstrating its exceptional analytical performance in detecting cysteine (Table 1). Furthermore, the proposed PEC sensor showed outstanding advantages in simple structure, convenient operation, economy and fast detection.

### 2.5. Reproducibility, Stability, and Selectivity

**Figure 5:**
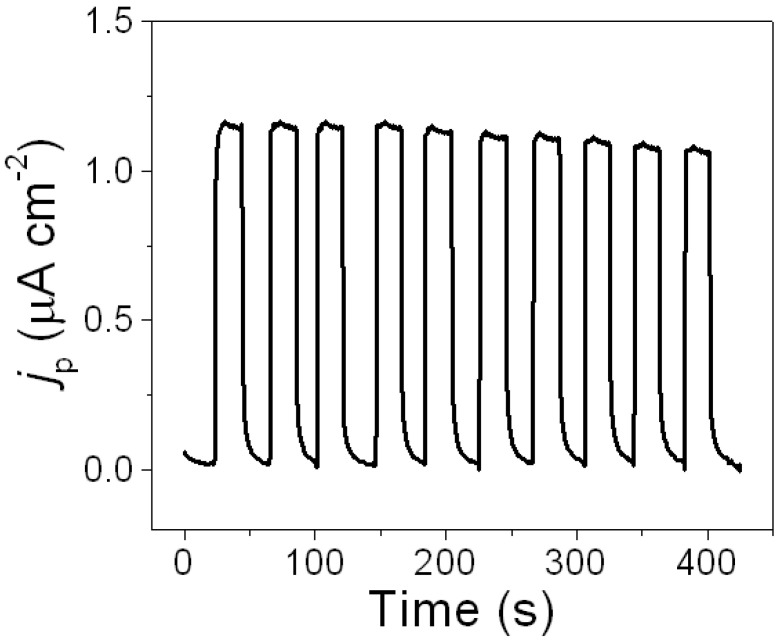
Time-based photocurrent response of the sensor under ten on/off irradiation cycles. The PEC measurements were carried out in 0.1 M PB (pH 7.0) containing 10 μM cysteine at applied potential of 0.0 V under the irradiation of a xenon light source.

To assess the reproducibility, six sensors were prepared independently and applied for detecting cysteine at a concentration of 10 μM. The present evaluation yielded an RSD of 3.58%, giving favorable repeatability. To examine the stability of the sensor, the photocurrent responses of the same concentration of cysteine on the same sensor were measured 10 times (Figure 5). The RSD of 4.31% suggests that the current response has good stability and the PEC sensor has desirable reusability.

**Figure 6:**
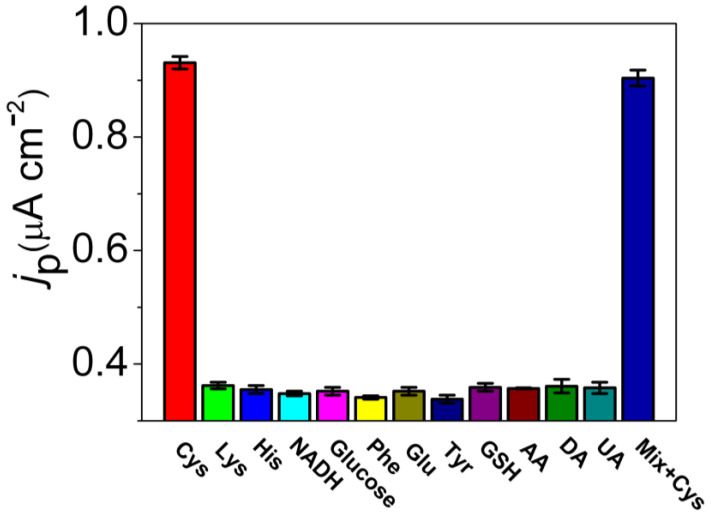
Photocurrent responses of the sensor towards 5 μM of cysteine and 50 μM of other interfering species. The PEC measurements were carried out in 0.1 M PB (pH 7.0) at applied potential of 0.0 V under light irradiation.

An interference experiment was conducted to evaluate the specificity of the sensor toward its target analyte. As displayed in Figure 6, eleven common interferences including lysine (Lys), histidine (His), phenylalanine (Phe), tyrosine (Tyr), glutamic acid (Glu), ascorbic acid (AA), uric acid (UA), glucose, dopamine (DA), glutathione (GSH), and nicotinamide adenine dinucleotide (NADH) had no significant influence on the photocurrent response of cysteine. The mixed solution of all interfering substances and cysteine brought out a negligible photocurrent change compared to the detection signal generated by cysteine alone. The corresponding raw data and figure are provided in the Supplementary Materials. As shown in the current vs. time data plot (Figure S1), the photocurrent curve of cysteine remained basically stable when a certain concentration of interfering substances was added to the cysteine sample, indicating that these introduced interferents have no response. The above results suggest that the proposed method has excellent selectivity for detecting cysteine, owing to the specifical recognition between cysteine and Au-ZnO@graphene QDs/ITO through the specificity of Au-S bonds.

### 2.6. Application in Real Sample Analysis

The reported cysteine concentration in human clinical fluids including serum, urine, and intracellular fluid is usually in the range of 30–200 μM [45]. The proposed sensor has a measurement range of cysteine concentration that is sufficient for clinical fluid detection. To assess the feasibility of the PEC sensor on detecting actual samples, the amount of cysteine in human urine and serum was measured. Human serum samples collected from a community hospital were diluted by 20-fold for detecting the photocurrent response of the PEC sensor. Human urine samples obtained from two healthy volunteers were immediately detected via an analysis after a 10-fold sample dilution. The standard addition technique was used for quantitative analysis. The results in Table 2 display that the average recoveries varied from 98.7 to 101.6%, demonstrating the developed strategy can be successfully used for complex real sample analysis. The cysteine concentration in urine and serum detected via this method were both reasonably consistent with the results reported in the literature [46], definitely indicating the method’s practicability in clinical fluids detection.

## 3. Materials and Methods

### 3.1. Chemicals

XFNANO Materials Tech Co. Ltd. (Nanjing, China) supplied graphene oxide (GO) with a diameter ranging from 0.5 to 5 μm, a thickness of 0.8 to 1.2 nm, a single layer ratio exceeding 99%, and a purity level surpassing 99%. Sinopharm Chemical Reagent Co., Ltd. (Shanghai, China) provided zinc acetate dihydrate, *N*,*N*-dimethylformamide (DMF), chloroauric acid, cysteine, lysine, histidine, phenylalanine, glutamic acid, tyrosine, sodium citrate, glucose, glutathione, ascorbic acid, uric acid (analytical grade). Furthermore, 0.1 M KH_2_PO_4_ and 0.1 M Na_2_HPO_4_ stock solutions were combined to prepare the phosphate buffer (PB).

### 3.2. Apparatus

D8-Advance X-ray diffractometer (Bruker AXS, Karlsruhe, Germany), UV-2600 UV-visible spectrophotometer (Shimadzu, Kyoto, Japan), JEM-2100 transmission electron microscope (JEOL, Tokyo, Japan), and FLS920 fluorescence spectrometer (Edinburgh, Britain) were used to characterize the prepared nanomaterials. PEC experiments were performed on a homemade PEC system, which included a 350 W xenon lamp as the irradiation source and a LK3200A electrochemical workstation (LANLIKE, Lanxi, China) for photocurrent detection.

### 3.3. Preparation of ZnO@graphene Quantum Dots

The core–shell nanocomposite was prepared via the chemical synthesis method after some modification for the previous literature [35]. Briefly, GO suspension (1 mg/mL) was prepared by dispersing GO in DMF under sonication for 10 min. Zinc acetate solution was obtained by dissolving 0.23 g of Zn (CH_3_COO)_2_·2H_2_O in 50 mL DMF. After gradually adding the GO suspension to the stirred zinc acetate solution, the resulting mixture was refluxed continuously at 95 °C for 5 h. Finally, the product was centrifuged and washed thoroughly with absolute ethanol and ultrapure water. The grayish white powder was obtained after vacuum drying for 12 h. The uncoated ZnO QDs were also synthesized via the same method except that GO was not added.

### 3.4. Construction of PEC Sensor

Before its modification, the ITO slices (0.5 × 4 cm^2^) were sequentially ultrasonically cleaned in acetone and ethanol for 20 min. The AuNPs were synthesized by reducing hydrogen tetrechloroaurate with sodium citrate [38]. Au nanoparticle-sensitized ZnO@graphene core–shell quantum dots (Au-ZnO@graphene QDs) were prepared as follows: A stable suspension was prepared by ultrasonically dispersing 10 mg of ZnO@graphene QDs in 1.0 mL of doubly distilled water for 30 min. Then, Au-ZnO@graphene QDs nanocomposites were obtained by ultrasonicating the mixed solution for 30 min after adding 1 mL of AuNPs colloidal solution to the above suspension. Subsequently, 10 μL of the Au-ZnO@graphene QDs solution was coated on a clean ITO electrode (0.25 cm^2^) and then air-dried. The formed PEC sensor was noted as Au-ZnO@graphene QDs/ITO. For the sake of comparison, ZnO@graphene QDs/ITO was constructed via the same process.

### 3.5. Photoelectrochemical Detection of Cysteine

Photoelectrochemical detections were performed on a homemade PEC system, which included an irradiation source as the light excitation system and a electrochemical workstation for photocurrent detection. The detection system consisted of a photoelectrochemical cell and a data-processing system. The photoelectrochemical cell was composed of a traditional electrochemical three-electrode system. A three-electrode cell with a saturated calomel electrode, a platinum sheet electrode and a modified ITO electrode (0.25 cm^2^) was employed for PEC measurements. White light was generated by a xenon lamp with a power output of 350 W. The distance from the working electrode to the light source remained constant at 20 cm. The current–time curve method was applied to measure the photocurrent in pH 7.0 PB containing different concentrations of cysteine with an illumination interval of 20 s. During operation, the working voltage was set to 0.0 V. The experiment was conducted at ambient temperature.

## 4. Conclusions

In summary, on the basis of Au nanoparticle-sensitized ZnO@graphene core–shell quantum dots as photoactive materials, we constructed a novel photoelectrochemical biosensor with high sensitivity for cysteine detection. The nanocoating core@shell structure with a built-in electric field and strong surface plasmon resonance effect allowed the synthesized ternary nanomaterials to exhibit an excellent photoelectrochemical performance. The specifical recognition between cysteine and Au-ZnO@graphene QDs/ITO through the specificity of Au-S bonds enhanced the selectivity for cysteine detection. We afforded a simple, fast, selective, and ultrasensitive photoelectrochemical technique for sensing trace cysteine in clinical detection. The study provided a new approach for designing high-performance PEC sensing materials and exploring their applications in biological research.

## Figures and Tables

**Table 1:** Comparison of cysteine assay by various reported PEC sensors.

Working Electrode	Linear Range (μM)	Detection Limit (nM)	Reference
Nafion/CdS-MV/ITO	0.2–2.8	100	[10]
Au-SnO_2_/CdS/ITO	0.4–120	100	[11]
PTh/TiO_2_/FTO	10–800	12,800	[39]
CuO−Cu_2_O/GCE	0.2–10	50	[40]
CA-TiO_2_/FTO	2.0–100	650	[41]
ZnTAPc-Gr/ITO	0.25–113	11.4	[42]
ITO/g-C_3_N_4_/Au	10–40	9200	[43]
Bi_2_MoO_6_/TiO_2_	0.5–600	150	[44]
TiO_2_-Au-BiOI	0.8–200	70	[45]
Au-ZnO@graphene QDs/ITO	0.1–150	8.9	This work

**Table 2:** Real sample analysis and recovery rate test in human serum and urine samples (n = 6).

Sample	Original (µM)	Added (µM)	Found (µM)	Recovery (%)	RSD (%)
Serum 1	6.57	5.00	11.47	99.1	3.3
Serum 2	6.97	5.00	12.16	101.6	2.8
Urine 1	10.61	10.0	20.34	98.7	2.0
Urine 2	13.56	10.0	23.28	98.8	1.6

## Data Availability

The data presented in this study are available in article and Supplementary Materials.

## Supplementary Materials

The following supporting information can be downloaded at: https://www.mdpi.com/article/10.3390/molecules29051002/s1, Figure S1: The current vs. time data plot of the sensor.

## References

[1] Bertoldo J.B., Terenzi H.S., Huttelmaier G., Bernardes J.L. (2018). Posttranslational chemical mutagenesis: To reveal the role of noncatalytic cysteine residues in pathogenic bacterial phosphatases. Biochemistry.

[2] Kurniawan A.F., Kurniawan F., Gunawan S.H., Chou M., Wang J. (2019). Disposable electrochemical sensor based on copper-electro deposited screen-printed gold electrode and its application in sensing L-cysteine. Electrochim. Acta.

[3] Gazit V., Ben-Abraham R., Coleman R. (2004). Cysteine-induced hypoglycemic brain damage: An alternative mechanism to excitotoxicity. Amino Acids.

[4] Qian Q., Deng J.J., Wang D.L., Yang L.F., Yu P., Mao L.Q. (2012). Aspartic acid-promoted highly selective and sensitive colorimetric sensing of cysteine in rat brain. Anal. Chem..

[5] Wei L., Lu X., Kang X., Song Y. (2020). Determination of glutathione and cysteine in human breast milk by high-performance liquid chromatography with chemiluminescence detection for evaluating the oxidative stress and exposure to heavy metals of lactating women. Anal. Lett..

[6] Ummadi M., Weimer B.C. (2002). Use of capillary electrophoresis and laser-induced fluorescence for attomole detection of amino acids. J. Chromatogr. A.

[7] Lu S., Li Z., Fu X. (2021). Carbon dots-based fluorescence and UV–vis absorption dual-modal sensors for Ag^+^ and L-cysteine detection. Dyes Pigm..

[8] Li H., Ye L., Wang Y. (2017). A glassy carbon electrode modified with hollow cubic cuprous oxide for voltammetric sensing of L-cysteine. Microchim. Acta.

[9] Zare H.R., Jahangiri-Dehaghani F., Shekari Z., Benvidi A. (2016). Electrocatalytic simultaneous determination of ascorbic acid, uric acid and L-cysteine in real samples using quercetin silver nanoparticles graphene nanosheets modified glassy carbon electrode. Appl. Surf. Sci..

[10] Long Y.T., Kong C., Li D.W. (2011). Ultrasensitive determination of cysteine based on the photocurrent of nafion-functionalized CdS-MV quantum dots on an ITO electrode. Small.

[11] Shen Q.M., Jiang J.Y., Liu S.L. (2014). Facile synthesis of Au–SnO_2_ hybrid nanospheres with enhanced photoelectrochemical biosensing performance. Nanoscale.

[12] Wang Y., Bian F., Qin X.F. (2018). Visible light photoelectrochemical aptasensor for chloramphenicol by using a TiO_2_ nanorod array sensitized with Eu (III)-doped CdS quantum dots. Microchim. Acta.

[13] Qin X.F., Geng L.P., Wang Q.Q., Wang Y. (2019). Photoelectrochemical aptasensing of ofloxacin based on the use of a TiO_2_ nanotube array co-sensitized with a nanocomposite prepared from polydopamine and Ag_2_S nanoparticles. Microchim. Acta.

[14] Son D.I., Kim T.W., Shim J.H. (2010). Flexible organic bistable devices based on graphene embedded in an insulating poly (methyl methacrylate) polymer layer. Nano Lett..

[15] Qin X.F., Geng L.P., Wang Q.Q., Shu X.L., Wang Y. (2019). A “signal-on” photoelectrochemical aptasensor based on graphene quantum dots-sensitized TiO_2_ nanotube arrays for sensitive detection of chloramphenicol. Talanta.

[16] Graeme W., Brian S., Kamat P.V. (2008). TiO_2_-graphene nanocomposites. UV-assisted photocatalytic reduction of graphene oxide. ACS Nano.

[17] Zhang X.Y., Li H.P., Cui X.L. (2010). Graphene/TiO_2_ nanocomposites: Synthesis, characterization and application in hydrogen evolution from water photocatalytic splitting. J. Mater. Chem..

[18] Chang H., Sun Z., Ho K.Y.F., Tao X., Yan F., Kwok W.M., Zheng Z. (2011). A highly sensitive ultraviolet sensor based on a facile in situ solution-grown ZnO nanorod/graphene heterostructure. Nanoscale.

[19] Huo P.W., Zhou M.J., Tang Y.F. (2016). Incorporation of N-ZnO/CdS/Graphene oxide composite photocatalyst for enhanced photocatalytic activity under visible light. J. Alloys Compd..

[20] Zhao X.M., Zhou S.W., Shen Q.M. (2012). Fabrication of glutathione photoelectrochemical biosensor using graphene-CdS nanocomposites. Analyst.

[21] Fu Y.S., Wang X. (2011). Magnetically separable ZnFe_2_O_4_-Graphene catalyst and its high photocatalytic performance under visible light irradiation. Ind. Eng. Chem. Res..

[22] Nipane S.V., Korake P.V., Gokavi G.S. (2015). Graphene-zinc oxide nanorod nanocomposite as photocatalyst for enhanced degradation of dyes under UV light. Ceram. Int..

[23] Zhang L.Z., Zhang J.N., Huang Y.H. (2021). Hexagonal ZnO nanoplates/graphene composites with excellent sensing performance to NO_2_ at room temperature. Appl. Surf. Sci..

[24] Niavol S.S., Khatibani A.B., Chauhanc D., Moghaddam H.M., Gao G.H. (2024). ZnO quantum dots decorated on graphene oxide and graphene nanoplatelets: Comparison the structure and sensing properties. Inorg. Chem. Commun..

[25] Low S.S., Michelle T.T., Loh H.S., Khiew P.S., Chiu W.S. (2016). Facile hydrothermal growth graphene/ZnO nanocomposite for development of enhanced biosensor. Anal. Chim. Acta.

[26] Hwa K.Y., Subramani B. (2014). Synthesis of zinc oxide nanoparticles on graphene–carbon nanotube hybrid for glucose biosensor applications. Biosens. Bioelectron..

[27] Zhang X., Zhang Y.C., Ma L.X. (2016). One-pot facile fabrication of graphene-zinc oxide composite and its enhanced sensitivity for simultaneous electrochemical detection of ascorbic acid, dopamine and uric acid. Sens. Actuators B Chem..

[28] Yukird J., Kongsittikul P., Rodthongkum N. (2018). ZnO@ graphene nanocomposite modified electrode for sensitive and simultaneous detection of Cd (II) and Pb (II). Synthetic Met..

[29] Shen Y.L., Yang J., Wan Q.J. (2022). Fabrication of a branch-like Ni-doped ZnO/graphene nanoplatelet composite for enhanced electrochemical determination of 17β-estradiol and acetaminophen. J. Electron. Mater..

[30] Li L., Zhai T.Y., Bando Y. (2012). Recent progress of one-dimensional ZnO nanostructured solar cells. Nano Energy.

[31] Mao Y.C., Yang H., Chen J.X. (2014). Significant performance enhancement of ZnO photoanodes from Ni (OH)_2_ electrocatalyst nanosheets overcoating. Nano Energy.

[32] Peng Y., Ji Y.J., Chen D. (2015). Ultrasound assisted synthesis of ZnO/reduced graphene oxide composites with enhanced photocata lytic activity and anti-photocorrosion. Appl. Surf. Sci..

[33] Liu F., Zhang Y., Yu J.H., Song X.R. (2014). Application of ZnO/graphene and S6 aptamers for sensitive photoelectrochemical detection of SK-BR-3 breast cancer cells based on a disposable indium tin oxide device. Biosens. Bioelectron..

[34] Yan Y., Ge Y., Mao H. (2023). Annealing temperature-dependent photoelectrochemical property of zinc oxide/graphene nanocomposite and the application for fabricating a “signal-off” photoelectrochemical aptasensing for ATP. IEEE Sens. J..

[35] Son D.I.B., Kwon W., Park D.H., Se W.S., Angadi B., Lee C.L., Choi W.K. (2012). Emissive ZnO–graphene quantum dots for white-light-emitting diodes. Nat. Nano..

[36] Bu Y.Y., Chen Z.Y., Li W.B., Hou B.R. (2013). Highly efficient photocatalytic performance of grapheme-ZnO quasi-shell-core com posite material. ACS Appl. Mater. Inter..

[37] Kim I., Bender S.L., Hranisavljevic J. (2011). Metal nanoparticle plasmon-enhanced light-harvesting in a photosystem I thin film. Nano Lett..

[38] Grabar K.C., Freeman R.G., Hommer M.B. (1995). Preparation and characterization of Au colloid monolayers. Anal. Chem..

[39] Wang Y., Wang W., Wang S., Chu W., Wei T., Tao H., Zhang C., Sun Y. (2016). Enhanced photoelectrochemical detection of L-cysteine based on the ultrathin polythiophene layer sensitized anatase TiO_2_ on F-doped tin oxide substrates. Sens. Actuators B Chem..

[40] Zhu Y.H., Xu Z.W., Yan K., Zhao H.B., Zhang J.D. (2017). One-step synthesis of CuO-Cu_2_O heterojunction by flame spray pyrolysis for cathodic photoelectrochemical sensing of L-cysteine. ACS Appl. Mater. Inter..

[41] Li F., Zhou B., Zhang W.B. (2018). A novel photoelectrochemical assay for cysteine based on the target-induced generation of photosensitizer. Int. J. Electrochem. Sci..

[42] Peng J.Y., Huang Q., Liu Y.X., Huang Y.Y., Xiang G. (2020). Photoelectrochemical detection of L-cysteine with a covalently grafted ZnTAPc-Gr-based probe. Electroanalysis.

[43] Li Y.R., Liu G., Ji D.Z., Zhang F.N., Liu Q.G. (2022). Smartphone-based label-free photoelectrochemical sensing of cysteine with cadmium ion chelation. Analyst.

[44] Liu W.T., Yao C.F., Cui H., Cang Y.G., Zhang Z.H., Miao Y.Q., Xin Y.M. (2022). A nano-enzymatic photoelectrochemical L-cysteine biosensor based on Bi_2_MoO_6_ modified honeycomb TiO_2_ nanotube arrays composite. Microchem. J..

[45] Xin Y.M., Wang Z., Yao H.Z., Liu W.T., Miao Y.Q., Zhang Z.G., Wu D. (2023). Au-mediated Z-scheme TiO_2_-Au-BiOI photoelectrode for sensitive and selective photoelectrochemical detection of L-cysteine. Sens. Actuators B Chem..

[46] Chen S., Gao H.L., Shen W.W., Lu C., Yuan Q.P. (2014). Colorimetric detection of cysteine using noncrosslinking aggregation of fluorosurfactant-capped silver nanoparticles. Sens. Actuators B Chem..

