# Supplementary material for: Surface Plasmon Resonance Enhanced Photoelectrochemical Sensing of Cysteine Based on Au Nanoparticle-Decorated ZnO@graphene Quantum Dots
Source: Molecules. 2024 Feb 25;29(5):1002. doi: 10.3390/molecules29051002 (PMC10935416; doi:10.3390/molecules29051002)
Supplement: Supplementary file 1 [file molecules-29-01002-s001.zip › molecules-2846063-supplementary.pdf]

Supplementary Materials

Surface plasmon resonance enhanced photoelectrochemical sensing of cysteine based on Au nanoparticle-decorated ZnO@graphene quantum dots

Jiaxin Liu, Fancheng Lin and **Yan Wang***

College of Chemistry, Chemical Engineering and Materials Science, Shandong Normal University, Jinan 250014, People's Republic of China

* Corresponding author. E-mail address: fagong@sdu.edu.cn (Y. Wang).

1. The interference experiment

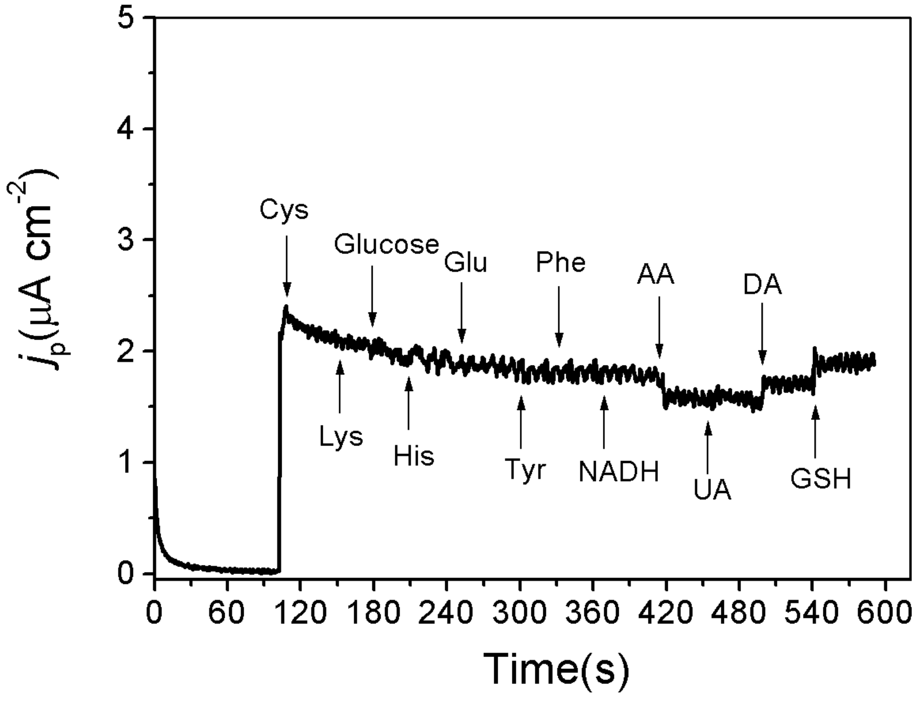

Figure S1. The current vs time data plot of the sensor towards 5 μM of cysteine and 50 μM of other interfering species. The PEC measurements were carried out in 0.1 M PB (pH 7.0) at applied potential of 0.0 V under light irradiation.
